# Metabolic dysfunction–associated steatotic liver disease and pregnancy

**DOI:** 10.1172/JCI186426

**Published:** 2025-05-15

**Authors:** Monika Sarkar, Tatyana Kushner

**Affiliations:** 1 Division of Gastroenterology and Hepatology, Department of Medicine, UCSF, San Francisco, California, USA.; 2 Department of Obstetrics & Gynecology, Department of Obstetrics and Gynecology, Weill Cornell Medicine, New York, New York, USA.

## Abstract

Metabolic dysfunction–associated steatotic liver disease (MASLD) is rising among reproductive-aged individuals and in pregnancy. MASLD in pregnancy does increase such risks as gestational diabetes, preeclampsia, and preterm birth. Although routine screening for MASLD has not been established in pregnancy, individuals with metabolic comorbidities, such as type 2 diabetes mellitus, should be evaluated by liver imaging and liver panel. Preconception counseling should address potential risks as well as need for optimized metabolic health before and during pregnancy. Fibrosis assessment should ideally be completed before pregnancy, to identify cases of cirrhosis that may warrant additional preconception management, such as variceal screening, as well as comanagement with maternal-fetal medicine specialists. In patients with MASLD, aspirin is advised at 12 weeks of gestational age to lower preeclampsia risk. In the absence of cirrhosis, no additional blood test monitoring is needed. In the general population, breastfeeding has beneficial effects on metabolic health in birthing parents and offspring and thus should be encouraged in the setting of MASLD, including access to enhanced lactation support. Research needs include evaluation of the long-term risks of MASLD in pregnancy on metabolic health in birthing parents and infants, as well as safety data for MASLD-directed therapies during pregnancy and lactation.

## Introduction

Metabolic dysfunction–associated steatotic liver disease (MASLD) is rising globally, including among reproductive-aged individuals, which confers unique risks during pregnancy and the postpartum period. In this Review, we address the epidemiology of MASLD, including distinct risk factors that contribute to MASLD in reproductive-aged female patients. We share suggested MASLD management strategies from the preconception through postpartum periods, including breastfeeding, as well as available data on the risks of MASLD in pregnancy to maternal and child health. Finally, we highlight existing knowledge gaps that should be prioritized for subsequent research.

Of note, the updated terminology of MASLD and metabolic dysfunction–associated steatohepatitis (MASH) is used throughout this Review (1), replacing prior terminology of nonalcoholic fatty liver disease (NAFLD) and nonalcoholic steatohepatitis (NASH), respectively. MASLD indicates the presence of hepatic steatosis with one or more cardiometabolic risk factors in the absence of other causes of hepatic steatosis, such as drug-induced or alcohol-related steatosis, for example. While the validation of this new terminology has not been conducted specifically in pregnant people with MASLD, there is more than 99% overlap between those identified as having NAFLD and those meeting criteria for MASLD within the general population (2).

## Epidemiology

MASLD prevalence is rising globally and expected to increase by 21%, to 100.9 million individuals by 2030. Importantly, the largest increase is evident in young adults under the age of 40 years (3, 4), and MASLD cirrhosis is now the leading indication for liver transplantation in women (5). This disease burden also extends to reproductive-aged female patients, who are experiencing rising rates of MASLD in pregnancy (6), in parallel to the rising rates of gestational obesity (7).

The largest study to date on MASLD in pregnancy leveraged the US National Inpatient Sample (NIS), evaluating over 5,500 pregnancies with MASLD identified by International Classification of Diseases (ICD) codes from 2007 to 2016. The prevalence of MASLD in pregnancy nearly tripled from 10.5/100,000 pregnancies to 28.9/100,000, which likely reflects an underestimation of disease given reliance on ICD codes at the time of hospital discharge (6). In a prospective study of 540 pregnant patients screened by liver ultrasound at a tertiary US academic center, MASLD prevalence was 19% among Hispanic patients, 17% among those with a body mass index (BMI) of 25 or higher, and 20% among those with a BMI of 30 or higher (8). However, less than 3% of these patients had documentation of MASLD prior to pregnancy, suggesting underdiagnosis before prenatal care (8). Studies from Korea have demonstrated MASLD prevalence in pregnancy of approximately 19% with the use of ultrasound, versus nearly 30% with the use of the fatty liver index (FLI; a formula using BMI, waist circumference, triglycerides, and γ glutamyl transpeptidase), whereas only 13% had MASLD when defined by the Hepatic Steatosis Index (HSI), which is calculated from the alanine aminotransferase/aspartate aminotransferase (ALT/AST) ratio, patient sex, and BMI (9–11). Although ICD codes may be more specific for MASLD diagnosis, these may not be entered by providers before the development of more significant liver disease. The specificities of noninvasive scoring systems, such as the HSI and FLI, range considerably, 25%–92% and 51%–93%, respectively, in the general population, and have not been validated in pregnancy (12–15). Imaging measures of MASLD, such as ultrasound, are less sensitive, specific, and reproducible than noncontrast MRI and liver biopsy at detecting mild steatosis, though their use is more feasible for pregnant patients (16).

Importantly, cirrhosis in pregnant patients is also rising, which confers increased risks to both birthing parents and their offspring (17, 18). In a large study from Ontario, Canada, evaluating cirrhosis in pregnancy, MASLD was the leading cause of liver disease, surpassing viral hepatitis, autoimmune liver disease, and other causes of chronic liver disease (19). Pregnant individuals with MASLD cirrhosis were also younger at conception and more likely to have obesity, dyslipidemia, and hypertension than those with non-MASLD causes of cirrhosis (19). Interestingly, they were also less likely to have had prior liver decompensation, which may translate to lower likelihood of cirrhosis diagnosis prior to pregnancy, underscoring the need for fibrosis assessment as part of routine preconception care of female patients with known MASLD.

## MASLD screening and evaluation in pregnancy

Though MASLD prevalence in pregnancy is rising, there are no routine screening recommendations specific to pregnant people. However, screening recommendations for the nonpregnant population should be applied for pregnant patients, if not previously conducted. The American Association for the Study of Liver Diseases (AASLD), European Association for the Study of the Liver (EASL), and American Gastroenterology Association (AGA) all advise MASLD screening in patients with type 2 diabetes mellitus (T2DM) or prediabetes, obesity, and at least one cardiometabolic risk factor (20–22). Polycystic ovary syndrome (PCOS) is an additional risk factor that is unique to young female patients, with nearly half of all with PCOS having concurrent MASLD (23) as well as an increased risk for MASH and advanced fibrosis (24, 25). While PCOS is not considered a criterion for MASLD screening in published guidelines, this condition typically manifests with other cardiometabolic risk factors that would merit MASLD screening. Previous or current gestational diabetes mellitus (GDM) is also strongly associated with prevalent MASLD (26–28); thus, MASLD screening is reasonable to consider in this patient population, particularly when this occurs in combination with other cardiometabolic risk factors, such as obesity.

As previously noted, the diagnosis of MASLD is based on the presence of steatosis evident on liver imaging or biopsy, with exclusion of nonmetabolic causes of hepatic steatosis and with the presence of at least one cardiometabolic risk factor (1). For patients at risk for MASLD, the AASLD, EASL, and AGA advise initial screening by way of Fibrosis-4 (FIB-4), which is calculated from age, AST, ALT, and platelet count (20–22). Despite the high prevalence of MASLD in the general population, the absolute risk of a liver-related event remains low, prompting MASLD screening recommendations to focus on detection of significant hepatic fibrosis. In the population of at-risk patients, FIB-4 is favored over other noninvasive fibrosis markers given its superior accuracy and its utility in predicting clinical outcomes (29–32). Vibration-controlled transient elastography (TE) can then be pursued for those with an indeterminant FIB-4 result (20–22).

Although FIB-4 is the most endorsed noninvasive fibrosis measure, it is less accurate in younger adults, a consideration relevant to the reproductive-aged female population (33). Otherwise, pregnancy does not influence the remaining components of this scoring system, such as aminotransferases, nor should platelet count decline to below clinically significant levels with pregnancy (34). Use of TE was also approved in pregnancy by the Food and Drug Administration (FDA) in 2023, thus providing another feasible option that can assess both presence and severity of steatosis and fibrosis. Of note, data from women without liver disease do show a slight increase in liver stiffness from preconception to pregnancy, and liver stiffness has been shown to increase somewhat over the course of pregnancy, likely in relation to increased portal blood flow (35, 36). Importantly, such changes should not affect the negative predictive value of TE in excluding advanced fibrosis/cirrhosis in pregnant patients with MASLD. Other serum markers of fibrosis that are used in general clinical practice are the AST/platelet ratio and the NAFLD fibrosis score, the latter composed of age, hyperglycemia, BMI, platelet count, albumin, and AST/ALT ratio. The NAFLD fibrosis score should not be used during pregnancy given the expected increase in BMI and dilutional decline in albumin levels. Regarding timing of fibrosis assessment, this should ideally be conducted before pregnancy in patients with MASLD, as those with cirrhosis warrant dedicated counseling regarding pregnancy risks, potential need for variceal screening, as well as multidisciplinary management with maternal-fetal medicine (MFM) specialists (34).

## Influence of pregnancy-related conditions on future MASLD risk

A growing body of literature highlights the influence of pregnancy and postpartum conditions on subsequent MASLD risk in both birthing parents and their offspring. Global data from Europe, Australia, and the United States cumulatively demonstrate an increased risk of MASLD in children, adolescents, and adults born to mothers with pre-pregnancy and gestational obesity (37–40). A Swedish study leveraging data with biopsy-confirmed MASLD in adults also found an independent risk for hepatic fibrosis and cirrhosis among those patients with MASLD born to mothers with overweight or obesity during pregnancy (41). Such findings underscore the relevance of dietary counseling in pregnant patients with elevated BMI, particularly as obesogenic diets during pregnancy contribute to later risk for overweight/obesity in offspring (42).

Incident MASLD in birthing parents is also influenced by pregnancy and postpartum contributors. Beyond gestational obesity, history of GDM has been independently associated with MASLD risk in midlife, conferring a more than 2-fold higher odds of hepatic steatosis by computed tomography (28). A recent Korean study evaluated the association of adverse pregnancy outcomes (APOs) defined as hypertensive disorders of pregnancy, GDM, preterm birth, low birth weight, and placental abruption with incident MASLD postpartum (43). MASLD diagnosis was defined by FLI at any point within 1 year after delivery. Although presence of at least one APO was associated with postpartum FLI, it is not clear to what degree components of the FLI (such as BMI, waist circumference, and triglycerides) reflect expected anthropometric and lipid changes in the initial postpartum period as opposed to true liver disease. In contrast, longer duration of lactation, particularly at least 6 months in duration, has been shown to protect against incident MASLD in midlife (44), and for birthing parents diagnosed with biopsy-confirmed MASLD later in life, longer duration of lactation is associated with decreased fibrosis severity (45).

## Metabolic adaptations of pregnancy and their influence on MASLD

**Figure 1:**
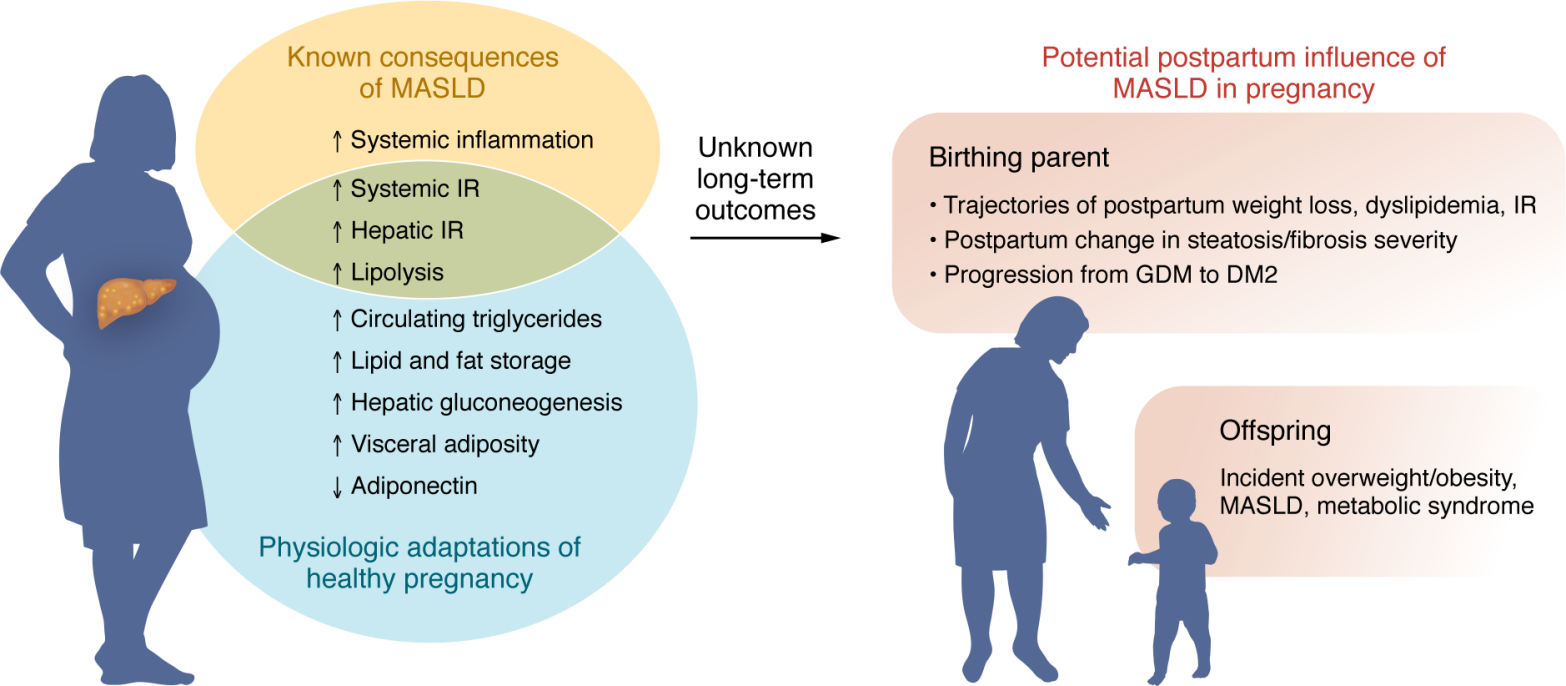
The intersection of maternal MASLD and physiologic adaptations of pregnancy. The influences of expected metabolic changes of pregnancy on postpartum metabolic health in mothers with MASLD and their offspring have not been delineated. DM2, type 2 diabetes mellitus; GDM, gestational diabetes mellitus; IR, insulin resistance; MASLD, metabolic dysfunction–associated steatotic liver disease.

During pregnancy, key metabolic adaptations occur to support a growing fetus, including an increase in visceral adiposity, lipolysis, triglyceride production, and decrease in insulin sensitivity (46). Placentally derived hormones, such as estrogen, progesterone, and growth hormone, play a key role in mediating these metabolic changes, promoting an anabolic state marked by increase in lipid storage during the first and second trimesters (47). As the fetus is unable to support gluconeogenesis, placenta-mediated release of prolactin, progesterone, and cortisol promotes insulin resistance, primarily within maternal skeletal muscle and adipose tissue. During the third trimester, there is a shift to a more catabolic state, with increase in hepatic gluconeogenesis, continued insulin resistance, and increase in maternal lipolysis (47). Physiologic adaptations in pregnant people with MASLD are clinically relevant, as visceral adiposity, dyslipidemia, and insulin resistance are risk factors for MASLD progression outside the context of pregnancy (22) (Figure 1). Moreover, insulin resistance is present in nearly all patients with MASLD (48), and the consequences of preexisting insulin resistance in the setting of pregnancies with MASLD could exacerbate underlying liver disease. While metabolic alterations of pregnancy improve after delivery, obesity in pregnancy and GDM do increase the risk for T2DM, indicating that preexisting metabolic disease influences the long-term trajectory of postpartum metabolic health (49). As discussed below, maternal MASLD is independently associated with metabolic complications during pregnancy, including hypertensive changes of pregnancy and GDM. However, metabolic outcomes beyond delivery have not been studied, reflecting an unmet research need given the potential clinical implications of metabolic changes of pregnancy on long-term liver health (Figure 1).

MASLD is present in nearly 80% of individuals with obesity (50), and robust data from the obesity literature do support the clinical relevance of gestational obesity to adverse cardiometabolic health of birthing parents and their offspring. The developmental origins hypothesis postulates that in utero and early life exposures result in sustained reprogramming of metabolic pathways that leads to susceptibility to chronic disease that extends long past the fetal and infant exposure (51). Gestational obesity appears to promote mesenchymal stem cells from umbilical cord blood to preferentially differentiate into adipocytes (51). Maternal obesity, GDM, and excess nutrition during pregnancy also lead to excess fetal exposure to lipid and glucose levels, which contribute to sustained alterations in infant metabolism (38, 52–54). In the background of genetic susceptibility, other epigenetic exposures, such as formula use prior to 6 months of age, increase risk for obesity in childhood, as well as MASLD in adolescence (38, 52–55). The compounding impact of MASLD during pregnancy on obesity-related stressors warrants evaluation because of the vital role of the liver in regulating glucose and lipid metabolism in humans. Beyond its effects in the liver, other MASLD-associated outcomes, including peripheral insulin resistance, systemic inflammation, and dysregulated lipid metabolism (56, 57), may help explain the observed increased risk of large-for-gestational age (LGA) infants being born to mothers with MASLD (6).

## Maternal and perinatal outcomes

Pregnancies with MASLD have been independently associated with adverse outcomes in birthing parents and infants. These findings are summarized in Supplemental Table 1, though it is important to highlight that variability in study populations and the method of defining MASLD contributes to heterogeneity in observed estimates (6, 9–11, 58–65). GDM remains the most studied outcome in relation to MASLD, with most data supporting a higher prevalence of GDM in pregnancies with MASLD, ranging from 20% to 30%, as well as an increased risk of GDM upon adjusted analyses (6, 10, 58, 59, 63, 65). In a prospective cohort study from Sri Lanka, the risk for GDM was associated with severity of ultrasound-graded hepatic steatosis (66). Given limited postpartum follow-up, it is not known whether MASLD increases the risk for progression from GDM to T2DM, which is an established long-term risk for pregnancies affected by obesity and GDM (67).

Studies evaluating hypertensive outcomes vary in their definitions, from inclusion of gestational hypertension alone or preeclampsia alone, to broader composite outcomes, including eclampsia and hemolysis, elevated liver enzymes, and low platelet (HELLP) syndrome. Across definitions, the risk for hypertensive complications does appear to be higher with MASLD. In the large US study using the NIS database, the odds of hypertensive complications defined as preeclampsia, eclampsia, or HELLP syndrome was more than 3-fold higher in pregnancies with MASLD than in pregnancies with no liver disease or with other types of chronic liver disease, supporting a specific influence of MASLD (6). Such findings were apparent even after adjustment for GDM, age, multiple gestation, as well as coexisting diagnosis codes for other metabolic comorbidities. The NIS data are consistent with adjusted analyses from a Swedish cohort evaluating risk of preeclampsia in relation to MASLD in pregnancy, as well as a Korean cohort evaluating a composite of gestational hypertension or preeclampsia (9, 58). Data from a Chinese cohort also identified an increased risk for preeclampsia in patients with hepatic steatosis and normal BMI, supporting an independent role of MASLD that cannot be explained by residual confounding by weight (64). As such, all pregnant patients with MASLD should be considered for aspirin therapy, as discussed below, to lower preeclampsia risk.

Data evaluating neonatal outcomes have included preterm birth, fetal growth restriction, and LGA. Maternal MASLD does appear to be an independent risk factor for preterm birth (6, 64), which may explain observed findings of low birth weight identified in two studies (58, 64). In a seemingly dichotomous manner, maternal MASLD also confers an increased risk for LGA infants, independent of GDM and maternal obesity (6, 11). Such findings raise the question of potential long-term effects of maternal MASLD on offspring health, as emerging data demonstrate a strong association of extremes of birth weight on MASLD risk in adulthood (68, 69). A longitudinal study of birthing parents with and without MASLD followed infants to age 2 years and found MASLD to be associated with very preterm birth (<32 weeks) and neonatal jaundice, independent of race and ethnicity, maternal obesity, T2DM, and hypertension. However, no changes in infant growth up to age 2 years were identified (70). A large prospective study enrolling those with and without MASLD in the first trimester of pregnancy also noted a higher risk of miscarriages in pregnancies with MASLD (66). Pooling existing data, a 2022 systematic review and meta-analysis by El Jamaly et al. evaluated the association of MASLD with gestational hypertension (OR 1.83, 95% CI 1.03–3.26), preeclampsia (OR 2.43, 95% CI 1.46–4.04), GDM (OR 3.23, 95% CI 1.97–4.31), premature birth (OR 2.02, 95% CI 1.44–2.85), and LGA (OR 2.01, 95% CI 1.72–2.37) (71). There was substantial heterogeneity, and publication bias was not assessed due to inadequate number of studies. As more data emerge, inclusion of prospective studies with imaging-based steatosis assessment will add rigor to existing data. Use of machine learning may also be helpful in this population, similar to a recent study using machine learning to predict GDM in patients with fatty liver that incorporated a combination of imaging, risk factors, and laboratory data (72).

## MASLD management in pregnancy and postpartum

**Figure 2:**
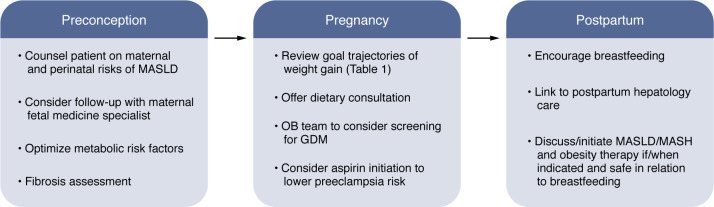
MASLD-specific management recommendations from preconception through delivery. GDM, gestational diabetes mellitus; MASLD, metabolic dysfunction–associated steatotic liver disease; MASH, metabolic dysfunction–associated steatohepatitis; OB; obstetric.

With the rising prevalence of MASLD in pregnancy, there is growing need for specific management recommendations from preconception through the postpartum period. The need for preconception counseling, including optimization of metabolic health prior to pregnancy, is endorsed by MASLD-specific recommendations by the AASLD and EASL (34, 73). These recommendations include the need for preconception counseling to review known maternal and perinatal risks associated with MASLD (Figure 2). Fibrosis assessment is advised before pregnancy to identify patients with cirrhosis, though it may be performed during pregnancy if not previously completed. As portal pressures increase during pregnancy, identification of cirrhosis has implications for variceal screening as well as an increased need for laboratory monitoring of liver function as pregnancy progresses. As cirrhosis confers additional risks during pregnancy and postpartum, such as hypertensive complications, postpartum infections, and bleeding (17, 18), patients should undergo preconception counseling, with need for multidisciplinary management with MFM specialists. In the absence of cirrhosis, routine monitoring of liver tests is not otherwise indicated in pregnant patients with MASLD (34, 73).

During pregnancy, patients should be guided on optimal ranges of weight gain per trimester, as detailed by the Centers for Disease Control and Prevention (Table 1) (74). This guidance is delineated by pre-pregnancy weight and presence of a singleton versus twin pregnancy. Maternal nutrition during pregnancy also affects health outcomes in the mother and infant and therefore should be optimized, with referral to nutritional consultation as needed. Indeed, the Colorado-based Healthy Start Study included detailed nutritional assessments in over 1,000 pregnancies and found higher maternal fiber and Mediterranean diet to confer lower MRI-quantified hepatic steatosis in children (75). Maternal intake of empty calories (a component of the Healthy Eating Index dietary assessment, which encompasses calories from solid fats, alcoholic beverages, and added sugars) was also associated with severity of hepatic steatosis in offspring, at a median age of 5 years. A small case-control study including 60 pregnant patients with GDM used ultrasound screening to identify 30 hepatic steatosis cases and 30 controls without steatosis. Cases and controls were matched on age, race, and BMI. The study observed notable dietary differences, including significantly higher carbohydrate intake and lower protein, fiber, and vitamin C intake, in those with liver disease (76), again supporting the need for dietary consultation in those with underlying MASLD.

Given the association of MASLD with GDM, earlier GDM screening can be considered (73). As MASLD is associated with hypertensive complications of pregnancy, aspirin use is often advised. While cesarean sections occur more commonly in patients with MASLD (58), mode of delivery should be guided by obstetric indications, rather than presence of liver disease alone (34, 73).

At present no medications are FDA approved for treatment of MASLD/MASH in pregnancy, and current clinical trials exclude pregnant populations. Though resmetirom (a thyroid hormone receptor agonist) is an FDA-approved medication for treatment of MASH with fibrosis, it is not approved for use in pregnancy (77). Vitamin E is also used for treatment of biopsy-confirmed MASH, though whether it is advisable to continue vitamin E during pregnancy is not clear. A Cochrane-based systematic review found vitamin E supplementation to be associated with increased risk of *term* prelabor rupture of membranes but not associated with premature rupture of membranes (78). Vitamin E was also protective against placental abruption and not associated with other maternal or perinatal outcomes. Thus, continuation of vitamin E during pregnancy is reasonable, particularly during the first and second trimesters, though it does warrant discussion with the patient regarding the potential risks and benefits later in pregnancy.

For weight loss, semaglutide (Wegovy), a glucagon-like peptide-1 (GLP-1) receptor agonist, and tirzepatide (Zepbound), a glucose-dependent insulinotropic polypeptide receptor and GLP-1 receptor agonist, are now FDA approved for treatment of obesity and actively being evaluated for treatment of MASLD/MASH (79, 80). Despite growing use of these agents among individuals of childbearing age, there are limited data on their use in pregnant people and neither is approved in pregnancy. Semaglutide discontinuation is advised at least 2 months prior to conception based on animal data demonstrating fetal anomalies (reduced growth and visceral abnormalities) and early pregnancy loss (81). Of note, doses administered in animal studies were significantly higher than the maximum recommended starting dose in humans, and drug exposure was limited to the first trimester. There are also limited data on semaglutide exposure via lactation, though more data are now being collected in the Wegovy Pregnancy Registry (82). Similarly, discontinuation of tirzepatide is advised at least 2 months prior to conception (though like semaglutide, full metabolism of tirzepatide is expected by 4 weeks since last use). Those with exposure to either drug in pregnancy are advised to report exposure information (81, 83). Similar to semaglutide, animal data on tirzepatide demonstrate increased incidence of external, visceral, and skeletal malformations when exposure occurred during organogenesis, with no data available on lactation (81). Thus, current guidance for patients with MASLD planning pregnancy is discontinuation before conception. In the interim, clinicians should await human registry data to determine the potential safety of these agents in later trimesters and during breastfeeding.

## Breastfeeding and MASLD

Data from mothers without MASLD have long demonstrated the benefits of breastfeeding, leading to recommendations by the American Academy of Pediatrics and World Health Organization to recommend exclusive breastfeeding for at least 6 months, with continued breastfeeding up to 2 years along with the introduction of solid foods (84). The protective effects of breastfeeding extend to metabolic outcomes in offspring, including lowered risk for childhood obesity and type 1 DM (85, 86). A growing body of literature has also shown that at least 6 months of breastfeeding lowers the risk for MASLD diagnosed in adolescents and young adults (38, 87). The potential mechanisms leading to reduced MASLD risk are multifactorial and include the possible promotion of favorable infant microbiota that influence infant metabolism and obesity (55). Breastfeeding is further linked to reduced levels of pro-inflammatory cytokines that are known to contribute to steatohepatitis (88–91). The mode of breastfeeding is also relevant, as receipt of breast milk by suckling versus passive bottle feeds promotes satiety response, leading to early programming of self-regulated eating that protects against childhood obesity (92, 93).

Outside the realm of MASLD, breastfeeding helps “reset” the aberrant physiologic changes of pregnancy in mothers (94). These metabolic benefits include more rapid return to pre-pregnancy weight and improvement in insulin sensitivity and serum lipid levels (94, 95). Longer duration of breastfeeding lowers the risk for T2DM in mothers with GDM, while breastfeeding duration of at least 6 months lowers the risk for incident MASLD later in life (44, 96, 97). Severity of MASLD may also be influenced by breastfeeding, as recent data show a protective effect of at least 3 months of cumulative breastfeeding against presence of advanced MASLD fibrosis in later life (45). However, these protective effects did not persist in women over age 50 at the time of liver biopsy, supporting the concept that chronologic aging has a stronger contribution to MASLD progression than breastfeeding. At present, no studies have evaluated the influence of breastfeeding among pregnancies with MASLD on either postpartum metabolic trajectories or MASLD progression. However, given the established metabolic benefits of breastfeeding in pregnancies affected by related comorbidities, such as T2DM and obesity, aggregate outcomes suggest that breastfeeding should be encouraged in postpartum mothers with MASLD when feasible, including optimization of lactation resources.

Addressing barriers to lactation is particularly relevant, as breastfeeding rates are notably lower among birthing parents with obesity, with potential clinical implications for birthing parents with MASLD (98–100). The reasons for lower breastfeeding rates among patients with obesity include factors that are commonly encountered with MASLD, including psychosocial stressors, prevalent T2DM (which affects milk supply), pre-term delivery, and unscheduled cesarean section (98). As we await more data on potential benefits of lactation to liver health, dedicated effort to support lactation in this high-risk population should be prioritized. As noted above, registry data are being collected for FDA-approved agents for MASLD and obesity, including resmetirom, semaglutide, and tirzepatide, in both pregnancy and lactation.

## Future directions

Despite the marked rise of pregnancies with MASLD, there remain no data on long-term clinical outcomes for birthing parents with MASLD or their offspring (Figure 1). Whether the expected metabolic changes of pregnancy hasten the progression of existing MASLD or its related metabolic consequences warrants evaluation. Dedicated interventional studies are also needed in this high-risk population, including evaluation of dietary and exercise interventions during pregnancy that may optimize nutrition to the mother and growing fetus, while mitigating progression of steatosis. The influence of pregnancy on steatosis severity through pregnancy and postpartum will be of benefit to elucidate longitudinally, including the specific effects of breastfeeding on trajectories of metabolic profiles in the postpartum period for patients with MASLD. As the intersection of reproductive health and liver disease garners attention (101), our field should anticipate burgeoning data to address these unanswered questions, which will no doubt enhance MASLD-specific management from preconception through delivery.

## Supplementary Material

Supplemental data

## Figures and Tables

**Table 1:**
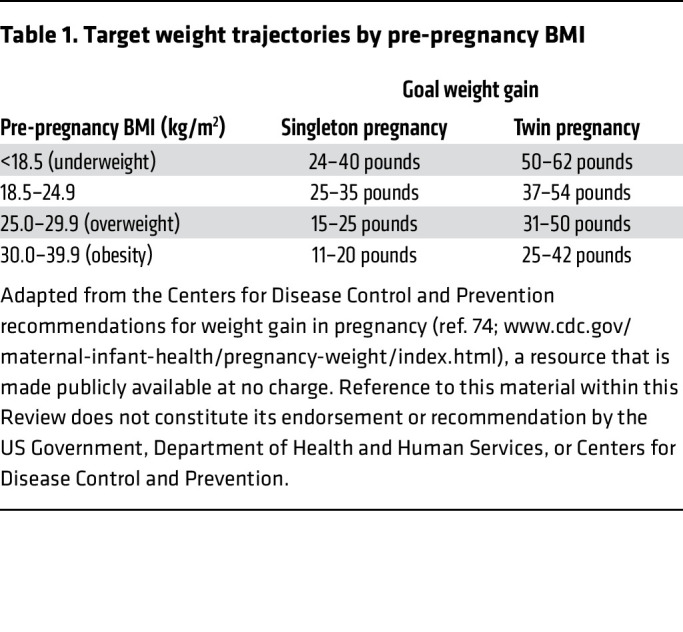
Target weight trajectories by pre-pregnancy BMI

## Version 1 05/15/2025

Electronic publication
