# Supplementary material for: Metabolic dysfunction–associated steatotic liver disease and pregnancy
Source: J Clin Invest. 2025 May 15;135(10):e186426. doi: 10.1172/JCI186426 (PMC12077888; doi:10.1172/JCI186426)
Supplement: Supplemental data [file jci-135-186426-s139.pdf]

Supplemental Table 1. Studies Evaluating Maternal and Perinatal Outcomes in Pregnancies With MASLD

Study	Country	Study design	MASLD prevalence	n	MASLD assessment	Pre-eclampsia (PE)/ GHTN	GDM	Neonatal outcomes	Covariates in adjusted models
Hagstrom et al. (Liver International 2016)[58]	Sweden	Retrospective cohort	0.006%	110 MASLD vs 1.9 million non-MASLD, non- PCOS controls	ICD-9 and ICD-10 codes for	PE: aRR 1.95 (95% CI 1.28-4.55)	aRR 2.78 (95% CI 1.25-6.13)	Low birth weight (aRR 2.40 (95% CI 1.21-4.78); Preterm birth (aRR 2.5 (1.38-5.55)	GDM: age, birth year, smoking, BMI<br><br>Other outcomes: age, birth year, maternal smoking, BMI
De Souza et al. (American Journal of Gastroenterology 2016)[59]	Ontario, Canada	Prospective cohort	17.4%	83 MASLD, 393 non-MASLD	US at 11-14 weeks gestation	NR	AOR 2.2 (1.1-4.3) for composite of impaired fasting glucose, impaired glucose tolerance, or GDM	NR	GDM: age, ethnicity, first-degree relative with DM2, BMI, change in BMI
Mousa et al. (British Journal of Biomedical Science 2018)[60]	Egypt	Prospective cohort	NR	200 MASLD, 200 non-MASLD controls	US in 1st trimester	PE: 25% vs. 14% (p=0.001)	GDM: 33% vs. 10% GDM (p=0.001)	Preterm birth: 11% vs 8% (p=0.31); Fetal growth restriction: 13% vs 10% (p=0.35)	NR
Herath et al. (Plos One 2019)[62]	Sri Lanka	Cross-sectional	18.2%	104 MASLD, 469 non-MASLD controls	US at delivery admission	AOR 2.09 (95% CI 1.07-4.10) for GHTN or PE	Incident DM in pregnancy or GDM: 27.2% vs. 17.5% (p<0.05)<br><br>AOR 1.30 (0.75-2.27) for incident DM in pregnancy or GDM	Preterm birth: 16.3% vs 12.4% (p=0.28)<br><br>Low birth weight: 18,4% vs 21.4% (p=0.77)	Incident DM or GDM: BMI >25, age >35, GHTN/PE<br><br>PE or GHTN: BMI >25, age >35, incident DM in pregnancy or GDM

Lee et al. (Diabetologia 2019)[10]	Korea	Prospective cohort	18.4% by US<br><br>28.5% by intermediate or higher FLI<br><br>12.0% high risk steatosis by HSI > 36	112 MASLD, 496 non-MASLD controls	US, FLI, HSI at 10-14 weeks	NR	AOR 6.66 (95% CI 2.26- 13.4) using MASLD by US	NR	GDM: Age, prior GDM, WC, hypertension, selenoprotein P, adiponectin
Lee et al. (Plos One 2019)[11]	Korea	Cohort	18.9% by US (any steatosis<br><br>4.5% by US (grade 2 or 3 steatosis)<br><br>29.2% by intermediate or higher FLI	108 MASLD per US, 32 high risk per FLI, 483 non MASLD controls	US at 10-14 weeks	NR	NR	LGA:<br>AOR 3.16 (1.16-8.59) using grade 2/3 steatosis on US	LGA: Age, BMI, GDM, triglycerides, HOMA-IR
Sarkar et al. (Journal of Hepatology 2020)[6]	U.S.	Cohort	0.02%	5640 MASLD, 115,210 other CLD, ~18 million no CLD	ICD-9 and ICD-10 codes at delivery discharge	16% in MASLD vs 4% in no CLD (p≤0.01); AOR 3.13 (2.61-3.75) for composite of pre-eclampsia, eclampsia, or HELLP (similar results vs no CLD and vs other CLD)	22% in MASLD vs. 7% in no CLD (p≤0.01); MV not performed	Preterm birth: AOR (vs no CLD) 1.60 (1.27-2.02); Fetal growth restriction: AOR (vs other CLD) 0.42 (0.25–0.71); LGA: AOR (vs other CLD) 1.84 (1.36–2.48)	All outcomes: Age, race, multiple gestation preexisting DM, obesity, dyslipidemia (estimates consistent with addition of GDM to models)

Jung et al. (Liver International 2020)[9]	Korea	Secondary analysis of prospective cohort	15.6% by US<br><br>28% by intermediate or higher FLI<br><br>13% high risk steatosis by HSI > 36	137 MASLD, 470 non-MASLD controls	US. FLI and HSI at 10-14 weeks gestation	MASLD by US: AOR 4.77 (1.26-15.83); MASLD by FLI: AOR 1.03 (1.01-1.05); HSI: 1.10 (1.01-1.21). Estimates reflect combination of GHTN, PE, or eclampsia	NR	NR	PE/GHTN/eclampsia: Waist circumference, blood pressure, fasting glucose, selenoprotein P
Sattari et al. (Journal of Investigative Medicine 2020)[62]	US	Prospective cohort	14%	12 MASLD, 72 non-MASLD controls	US in 3rd trimester	NR	42% vs 61% (p=0.21)	NR	NR
Lee et al. (Clinical Gastroenterology and Hepatology 2022)[63]	Korea	Secondary analysis of multicenter prospective cohort	11.7% MASLD	178 MASLD, 1523 non MASLD controls	US in 1st trimester plus >/= 1 metabolic condition	AOR 2.69 (1.24-5.84)	AOR 4.22 (2.65-6.73)	LGA: 15% in vs 9% (p=0.019)	All outcomes: Age, prior GDM, family history DM2, prior hypertensive disease of pregnancy, chronic hypertension, pre-pregnancy WC
Koralegedara et al. (BMJ Open Gastroenterology 2022)[66]	Sri Lanka	Population-based prospective cohort study	51%	324 SLD, 308 non SLD	US in 1st trimester		Fatty liver grade (FLG) 2: RR 12.5 (2.2-66.4)	Early pregnancy miscarriage: FLG 2: aOR 4.2 (1.9-9.1)	GDM:<br>Age, famhx DM, parity, wait-to-hip ratio, female education, BMI<br>Miscarriages:<br>Age, parity, blood pressure, blood sugar, hemoglobin, BMI

Qian et al. (Journal of Clinical Endocrinology and Metabolism 2023)[64]	China	Retrospective cohort	3.8%	554 MASLD, 14,154 non-MASLD controls	US in 1st trimester	AOR GHTN 3.05 (95% CI 2.19-4.26); AOR PE or eclampsia 3.99 (95% CI 2.59-6.01)	AOR 2.48 (95% CI 1.89-3.25)	Preterm birth: AOR 2.16 (95% CI 1.47-3.18); Low birth weight: AOR (2.35, 95% CI 1.41-3.90)	All outcomes: age, ethnicity, education, gravidity, parity; AST and ALT
Jafari RM et al. (Gastroenterology and hepatology from bed to bench 2024)[65]	Iran	Prospective cohort	NR	180 MASLD; 180 non-MASLD controls	US (timing not reported)	16.1% vs 6.1% (p=0.003)	29% vs 13% (p<0.001)	Preterm birth: 18% vs 13% (p=0.25); Low birth weight 2.2% vs 1.7% (p=0.80)	NR

Abbreviations: AOR, adjusted odds ratio; aRR, adjusted risk ratio; A/w, associated with; BMI, body mass index; CLD, chronic liver disease; DM2, type 2 diabetes mellitus; FLI, fatty liver index; GDM, gestational diabetes; GTN, gestational hypertension; HSI, hepatic steatosis index; HELLP, hemolysis, elevated liver tests low platelets, HOMA-IR, homeostasis model assessment of insulin resistance; ICD, international classification of diseases; LGA, large for gestational age; MASLD, metabolic dysfunction-associated steatotic liver disease; MD, metabolic dysfunction; MV, multivariate analysis; NA, not applicable; NR, not reported; PE, pre-eclampsia; U.S. United States; US, ultrasound; WC, waist circumference

Reference numbers in "Study" column correspond to the main text reference list.
